# Experimental evaluation of stiffness predictions of multiaxial flax fibre composites by classical laminate theory

**DOI:** 10.1371/journal.pone.0234701

**Published:** 2020-06-24

**Authors:** Yosuke Ueki, Hans Lilholt, Bo Madsen

**Affiliations:** 1 Reliability Science Research Department, Center for Technology Innovation–Mechanical Engineering, Research & Development Group, Hitachi, Limited, Hitachinaka, Japan; 2 Section of Composite Materials / Section of Composite Mechanics and Structures, Department of Wind Energy, Technical University of Denmark, Kongens Lyngby, Denmark; Missouri University of Science and Technology, UNITED STATES

## Abstract

Despite the good mechanical properties of natural fibre composites, their use in load-bearing components is still limited, which may be due to lack of knowledge and confidence in calculating the performance of the composites by mechanical models. The present study is providing an experimental evaluation of stiffness predictions of multiaxial flax fibre composite by classical laminate theory (CLT). The experimental base is (i) multiaxial flax fibre composites fabricated with two types of biaxial non-crimp fabrics, having a nominal yarn orientation of ±45°, and (ii) uniaxial flax fibre composites fabricated with the same flax yarn as used in the fabrics. The fabricated composites are characterised by volumetric composition, yarn orientation and tensile properties. A fast and easy operational Fast Fibre Orientation (FFO) method is developed to determine the actual yarn orientation in fabrics and composites. It is demonstrated that the FFO method is a robust method, giving repeatable results for yarn orientations, and it can be used both on fabrics and composites. CLT predictions of stiffness of the multiaxial flax fibre composites are shown to be in good agreement with the measured stiffnesses of the composites in three testing directions (0°, 45°, and 90°). The use of the actual yarn orientations measured by the FFO method, instead of the nominal yarn orientations of ±45°, is shown to result in improved CLT predictions of stiffness with a mean deviation between predictions and measurements on 0.2 GPa. Altogether, it is demonstrated that stiffness of multiaxial flax fibre composites can be accurately predicted by CLT, without any fitting constants, based on independently determined stiffness parameters of the related uniaxial flax fibre composite, and based on measured yarn orientations in the flax fibre fabric.

## Introduction

Composite materials is an established class of materials widely used in industry for structural applications, such as wind turbine blades and automobile parts, requiring high stiffness and strength, coupled to low weight. Natural fibre composites, e.g. based on flax fibres, are emerging types of composites that are gaining increased interest in industry due to their good mechanical properties, and with lower weight than conventional composites [1–5]. The potential lower environmental impact of natural fibre composites [6] is a further argument for the interest in these materials.

The current industrial use of natural fibre composites is mostly limited to components with little load-bearing requirements, such as the body panels in automobiles [7]. One reason why natural fibre composites are still of limited use in load-bearing components may be due to the lack of knowledge and confidence in calculating the performance of the composites by mechanical models. The properties of uniaxial natural fibre composites, with fibres only in one direction, are well studied, and are shown to be well predicted by common micromechanical models [8, 9]. The properties of the more industrial relevant multiaxial composites, so-called composite laminates consisting of uniaxial layers of fibres, where the layers are oriented in different directions, are still to be evaluated and documented. In the case of conventional fibre composites, the classical laminate theory (CLT) is widely used and is considered to be the most accurate method for calculating the mechanical properties of composite laminates [10, 11].

Recently, tailor made flax fibre fabrics for composites are produced by a number of companies in Europe. The fabrics are so-called non-crimp fabrics, and typically with a biaxial nominal ± 45° fibre orientation. The laying up of these fabrics on top of each other is the basis for fabrication of multiaxial flax fibre composite laminates. In this context, the actual fibre orientation in the fabrics (which is likely to deviate from the nominal one) is of central importance for the resulting mechanical properties of the composites.

The present study aims to evaluate the use of CLT to predict stiffness of multiaxial flax fibre composites. Stiffness is a fundamental mechanical property for most structural applications. The evaluation of the CLT predictions will be based on experiments where two types of non-crimp biaxial flax fibre fabrics are used to fabricate multiaxial composites. In addition, uniaxial flax fibre composites are fabricated to provide input parameters for the CLT predictions. A fast and easy operational method is developed to determine the actual yarn orientation in fabrics and composites. The measured stiffness of the multiaxial composites, in three testing directions, is compared with the stiffness predicted by CLT.

## Materials and methods

### Biaxial fabrics, yarns and resin

**Fig 1:**
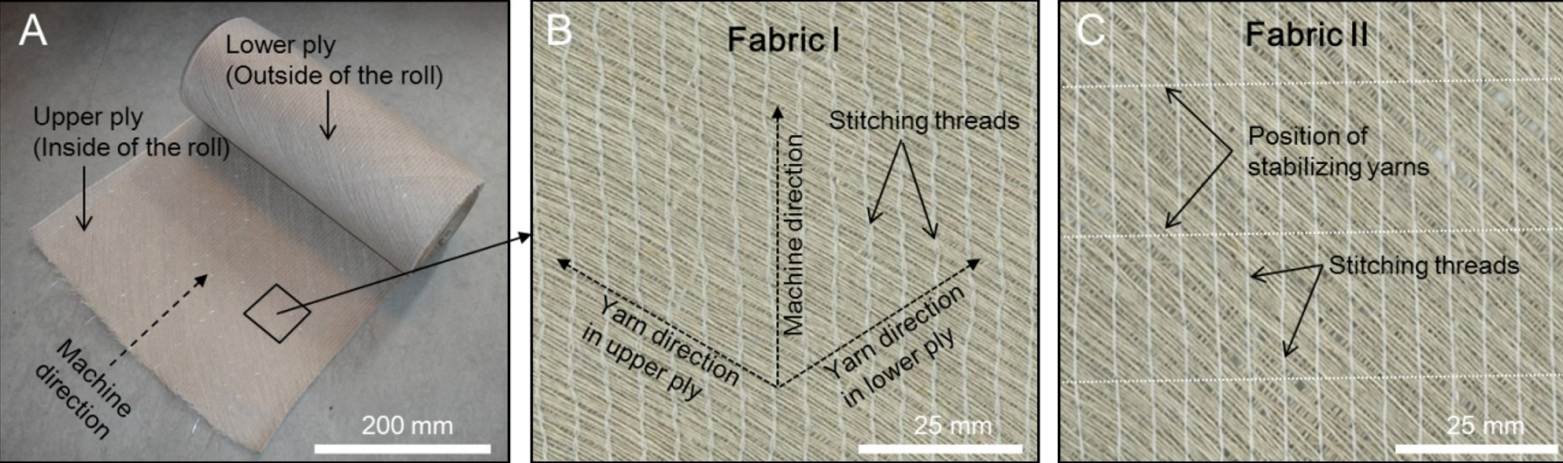
Photographs of the used non-crimp biaxial flax fibre fabrics. A: Roll with width of 330 mm. B, C: Magnified view of fabric I and fabric II.

Two types of commercial non-crimp biaxial flax fibre fabrics (AmpliTex 5008), purchased from Bcomp Ltd., Switzerland, are used. Specifications of the two fabrics and their photographs are shown in Table 1 and Fig 1. The two types of fabrics differ by whether or not a flax yarn, positioned a regular intervals of about 24 mm transverse to the machine direction, is used to stabilize the fabric. The two types of fabrics are hereafter referred to as “fabric I” (no stabilizing yarns) and “fabric II” (with stabilizing yarns). In addition to the biaxial fabrics, bobbins with the same type of flax yarn as used for the fabrics was purchased from Bcomp Ltd., and used for fabrication of uniaxial composite laminates. The fibre twisting angle in the flax yarn was estimated by optical microscopy to be approximately 15 degrees. As matrix in the composites, an epoxy resin system of Araldite LY1564SP (epoxy) and Araldite 3486(hardener) was used. The mixing ratio was set to be 100(resin): 34(hardener). The density, stiffness and Poisson’s ratio of the fully cured epoxy resin was measured to be 1.151 g/cm^3^, 3.0 GPa and 0.35, respectively.

**Table 1:** Specifications of the used non-crimp biaxial flax fibre fabrics.

Fabric		Area weight	350 g/m^2^
Constituents	Yarn	Type	Flax, 106 tex
		Orientation	± 45° (nominal)
	Stitching thread	Type	Polyester
		Area weight	6 g/m^2^
	Stabilizing yarn	Type	Flax, 106 tex
		Area weight	[none] (fabric I)
			4 g/m^2^ (fabric II)

### Fast fibre orientation method

**Fig 2:**
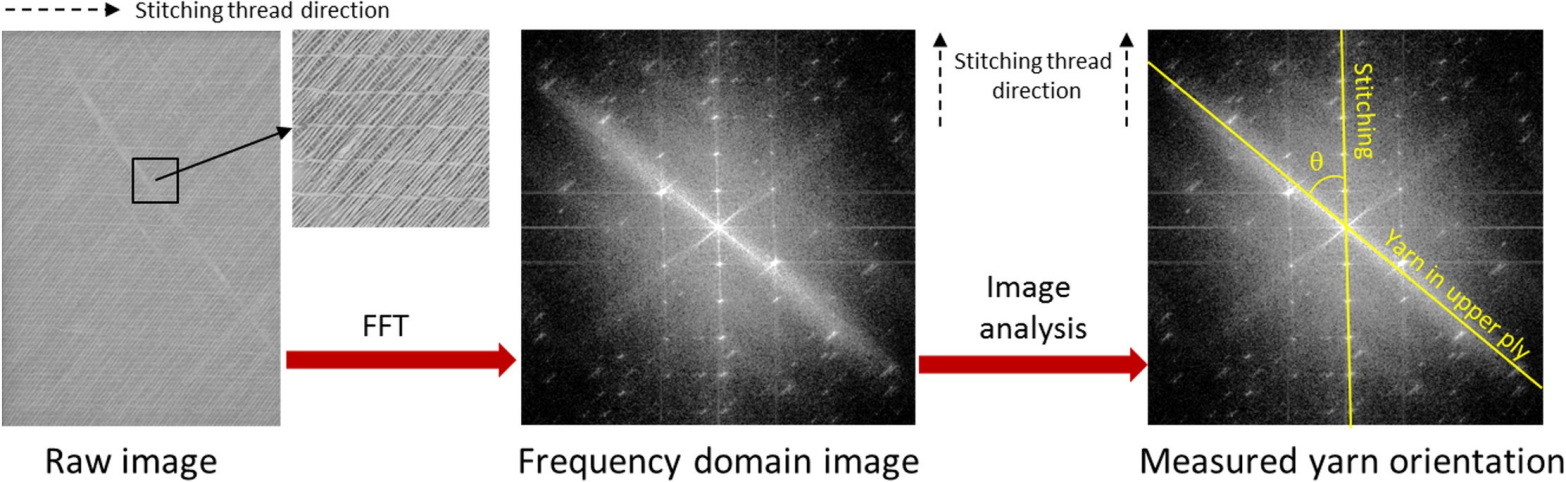
Illustration of the Fast Fibre Orientation (FFO) method for measurement of yarn orientation in a biaxial fabric using 2D Fast Fourier Transformation (FFT) and image analysis.

A fast and easy operational method was developed to accurately determine the orientation of yarns in fabrics and composites. The developed Fast Fibre Orientation (FFO) method is illustrated in Fig 2. The surface of a biaxial fabric sheet was first scanned by a standard flat head scanner (CanoScan9950F, Canon, Japan) using a reflecting illumination mode. Due to the size of the scanner, the scanned area was limited to A4 size. The scanned image was transformed into a frequency domain image by a 2D Fast Fourier Transformation (FFT) function using image analysis software (ImageJ 1.47v, National Institute of Health, USA). FFT recognizes an image as a superposition of spatially periodic patterns of frequencies and directions. Thus, for example, aligned yarns with an angle of 0° are recognized as a composition of periodic patterns with an angle of 90° and with a frequency corresponding to the distance between the yarns. In Fig 2, middle, the two oblique bright lines and the one vertical bright line correspond to the flax yarns in the two plies, and the stitching threads, respectively. The angles of these lines were measured manually using the ImageJ software. A fabric sheet was scanned on both sides, and the results from each scan were used to evaluate the yarn direction in the upper ply, only, as indicated in Fig 2, right. The direction of the stitching threads (corresponding to the machine direction) was used as the reference direction, and the angle between the stitching threads and the yarns was defined as the yarn orientation angle (θ). Yarns oriented to the left of the reference direction are presented as positive angles, and yarns oriented to the right of the reference direction are presented as negative angles.

### Fabrication of multiaxial composite laminates

**Fig 3:**
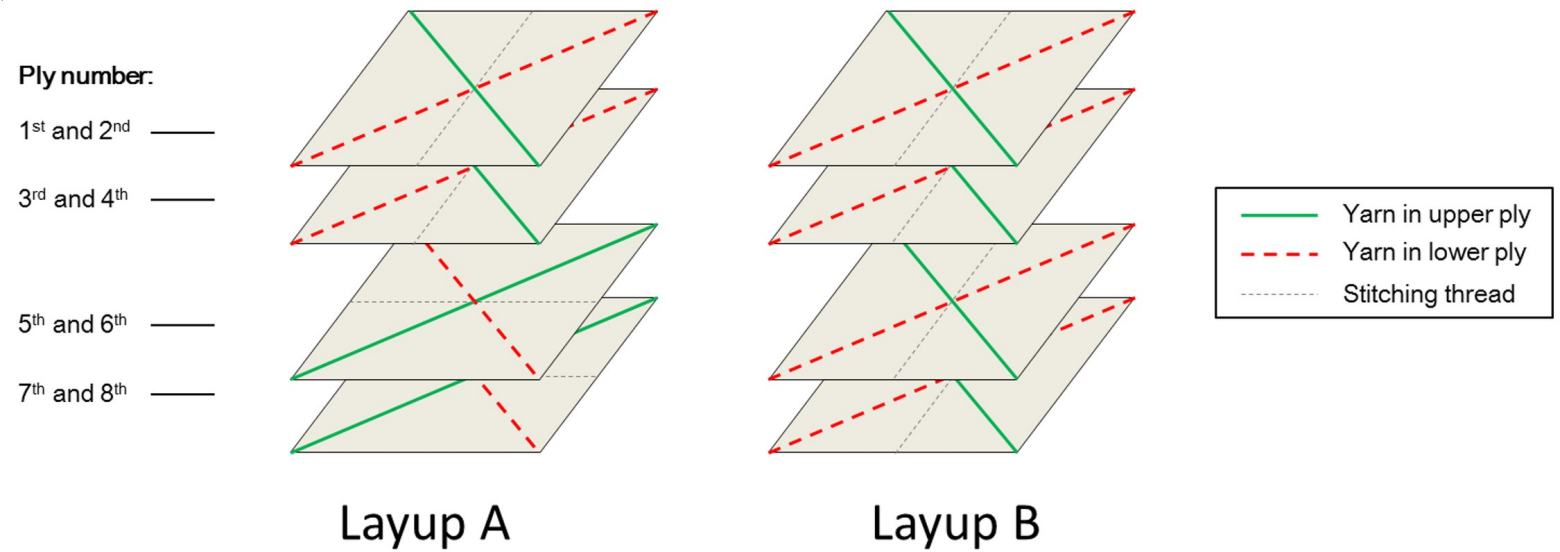
Schematic illustration of the layup configurations in the fabricated multiaxial composite laminates with layup A and layup B. The laminates are made from 4 sheets of biaxial fabric, each consisting of two plies, giving 8 plies in total. In layup A, the two bottom fabric sheets are rotated in plane with an angle of 90° to the two top fabric sheets. In layup B, the four fabric sheets are placed on top of each other, without any rotation.

As shown in Fig 3, multiaxial composite laminates with two different layup configurations, layup A and layup B, were fabricated from the biaxial fabrics. Four sheets of fabrics were used, and since each sheet consists of two plies, then in total, the composite laminates are consisting of 8 plies. In layup A, the two bottom fabric sheets are rotated in plane with an angle of 90° to the two top fabric sheets. In layup B, the four fabric sheets are placed on top of each other, without any rotation.

If the yarn orientation of the biaxial fabric is exactly ± 45°, then layup A gives a so-called symmetric laminate where the yarn angles around the centre thickness line of the laminate are symmetric to each other. This is a typical applied requirement for composite laminates to avoid that a fabricated laminate gets distorted, by balancing the internal stresses. Layup B gives a non-symmetric laminate. Furthermore, if the yarn orientation of the biaxial fabric is exactly ± 45°, the yarn orientation in the laminates made with layup A and B will be the same, and equal to ± 45°.

**Fig 4:**
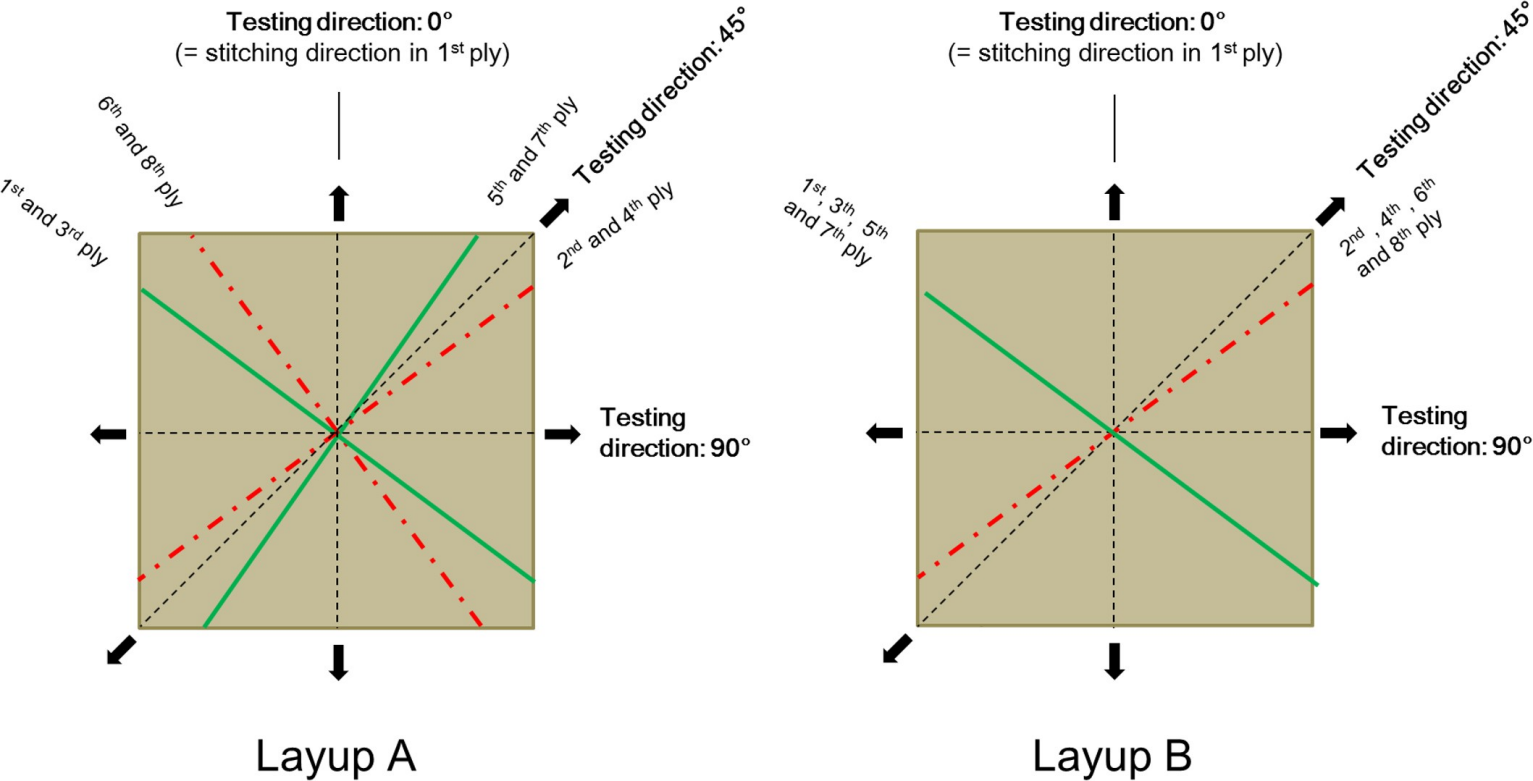
Schematic illustration of yarn directions in the multiaxial composite laminates with layup configurations A and B. The mechanical testing directions, 0°, 45° and 90° are also indicated. Shown is an example of a biaxial fabric with + and–yarn angles above 45°.

In contrast, if the yarn orientation of the biaxial fabric deviates from the nominal ± 45°, then both layup A and B give non-symmetric laminates. Furthermore, the yarn orientation in the laminates with layup A and B will be different from each other, and the yarn orientation will not be biaxial, but instead it will be multiaxial. This is illustrated in Fig 4.

Vacuum infusion was performed to impregnate the layups of fabric with epoxy resin, followed by curing (5 hours at 50°C), and then de-moulding of the composite laminates and post-curing (4.5 hours at 80°C). The dimensions of the fabricated laminates were 300 x 300 x 2.9 mm.

### Fabrication of uniaxial composite laminates

Fabrication of uniaxial composite laminates was performed by filament winding followed by vacuum infusion. The flax yarn was wound on an aluminium plate of 470 x 400 mm, and with a lateral movement per rotation of 1 mm. The resulting misalignment of the wound yarns is about 0.1° (= tan_-1_(1 mm/2 x 470 mm), and will be neglected. The number of winding layers was set to be 14 in order to obtain composite laminates with a thickness of 3.0 mm, i.e. the same thickness as the multiaxial laminates. After filament winding, the yarns on the aluminium plate were enveloped in a vacuum bag, and infused with epoxy resin and cured, resulting in two laminates per fabrication. The applied curing conditions were the same as the ones used for the multiaxial composite laminates.

### Volumetric composition in composites

The volume fractions of fibres (*V*_*f*_), matrix (*V*_*m*_), and porosity (*V*_*p*_) in composites can be calculated as follows:

$$V_{f}=\left(\frac{\rho_{c}}{\rho_{f}}\right)\times W_{f}$$ (1)

$$V_{f}=\left(\frac{\rho_{c}}{\rho_{m}}\right)\times \left(1-W_{f}\right)$$ (2)

$$V_{p}=1-V_{f}-V_{f}$$ (3)

where *ρ*_*f*_, *ρ*_*m*_ and *ρ*_*c*_ are the density of fibres, matrix and composite, respectively, and *W*_*f*_ is the fibre weight fraction. Thus, in addition to the density of fibres and matrix, measurements are required of the fibre weight fraction and the density of the composites. In the present study, to perform these measurements, 6 specimens with planar dimensions 25 x 25 mm were cut from the composite laminates. The edges of the specimens were polished to remove any loose fragments, and their in-plane dimensions (length and width) were measured with an accuracy of 0.01 mm. After drying under vacuum overnight, the weight of the specimens (*m*_*c*_) was obtained. By knowing the area of the specimens (*A*_*c*_) (= length x width), the weight of the fibres (*m*_*f*_) in the specimens was calculated from the area weight of the fabric, in the case of the multiaxial composites (Eq 4), or from the linear density of the yarn, in the case of the uniaxial composites (Eq 5):

$$m_{f}\left[kg\right]=Fabric\;area\;weight\left[\frac{kg}{m^{2}}\right]\times Number\;of\;fabric\;sheets\times A_{c}\left[m^{2}\right]$$ (4)

$$m_{f}\left[kg\right]=\left(Yarn\;linear\;density\left[\frac{kg}{m}\right]\times \frac{Number\;of\;winding\;layers}{Distance\;between\;yarns\left[m\right]}\right)\times A_{c}\left[m^{2}\right]$$ (5)

The fibre weight fraction, *W*_*f*_, was then calculated as the ratio between *m*_*f*_ and *m*_*c*_. To determine the composite density (*ρ*_*c*_), the specimens were weighed submerged in water, and the density was determined by utilizing the Archimedes principle. In a previous study by Madsen et al. [12], *ρ*_*f*_ for flax fibres was determined to be 1.540 g/cm^3^.

### Yarn orientation and tensile properties of composites

**Fig 5:**
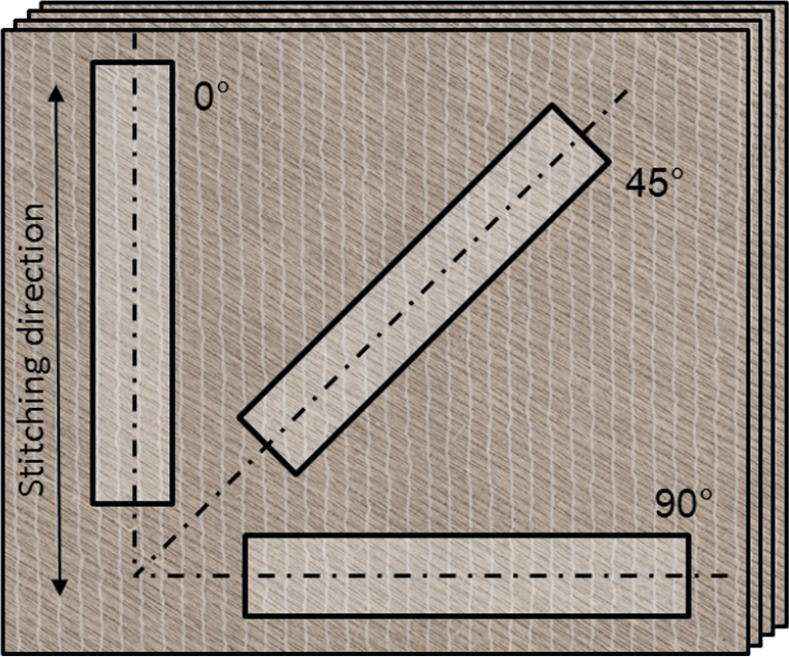
Cut out directions for tensile test specimens of multiaxial composite laminates.

For the multiaxial composite laminates, tensile test specimens with a rectangular shape (150 x 25 mm, with tabs) were cut in three directions of the laminates: 0° (parallel to the stitching threads in the upper fabric sheet), 45° and 90°, as shown in Fig 5. The relationship between these testing directions and the yarn orientations of the multiaxial laminates is shown in Fig 4. For the uniaxial composite laminates, tensile test specimens with a rectangular shape (180 x 25 mm, with tabs) were cut along the yarn direction.

**Fig 6:**
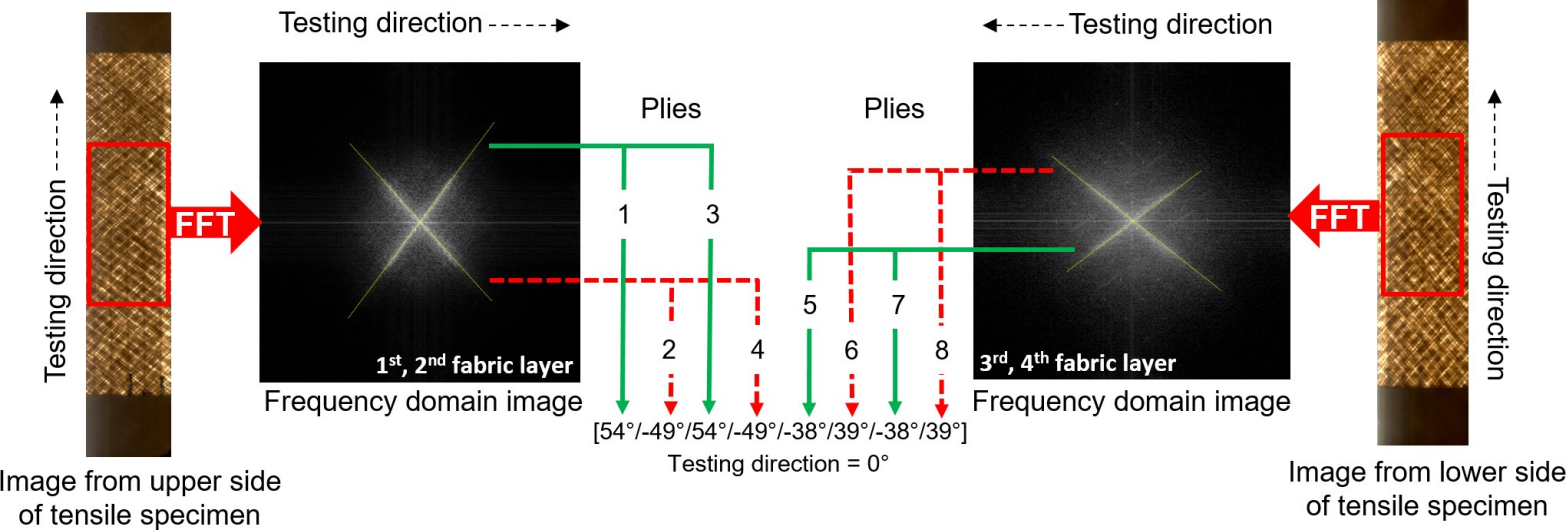
Illustration of the Fast Fibre Orientation (FFO) method for measurements of yarn orientation in a tensile specimen of a multiaxial composite. Shown is an example of a composite with fabric I, and with layup A.

Before tensile testing of the multiaxial composites, the yarn orientation in the tensile specimens was determined by the developed FFO method. The specimens were scanned by the flat head scanner using a transmitted illumination mode. Fig 6 shows an example of scanned images of the upper and lower side of a tensile specimen, and the corresponding frequency domain images where four bright lines can be seen in each image. It is assumed that the two oblique lines indicate the yarns in the two fabric sheets closest to scanned surface. The horizontal and vertical lines (which are not so clear in the image) indicate the stitching threads. It was not possible to distinguish between the two top fabric sheets in the images, and, as indicated by the extracted angles in Fig 6, it was therefore assumed that they have identical yarn orientations.

Tensile tests of the specimens were performed using a displacement rate of 2 mm/min on a hydraulic testing machine (Instron, USA) with a 100 kN load cell. For the multiaxial composites, 3 specimens were tested for each testing direction, and for the uniaxial composites, 5 specimens were tested. The lower number of tested specimens for the multiaxial composites was due to the limiting area of the laminates. Strain was measured by two extensometers with 50 mm gauge length attached to each side of the specimens. Stiffness was determined as the slope of the initial linear part of the measured stress-strain curves in the strain interval 0.01–0.15%.

### Prediction of composite stiffness

Preliminary, the stiffness of the multiaxial composite laminates was predicted by three methods: 1) by using the rule of mixtures model with the Krenchel fibre orientation factor [9, 13] to calculate a combined effect of all fibre orientations, 2) by using the analytical equation for off-axis loading of uniaxial composites [11], together with a mixtures relationship to calculate the contribution of the individual plies, and 3) by using the classical laminate theory (CLT) [10, 11] to take into account the existence of mechanical interaction between the plies. It was found that all three methods led to predictions which were in acceptable agreement with the experimental data. The CLT method, however, was consistently giving the best predictions, and therefore this method will be used in the present study to predict the stiffness of the fabricated multiaxial composite laminates.

A complete description of classical laminate theory (CLT) is found in [10, 11]. CLT is consisting of two steps of linear algebra calculations. Firstly, the stiffness of the uniaxial plies in the local coordinate system (with respect to the fibre direction) is transformed into the global coordinate system (with respect to the load direction) using the stiffness matrix (known as the *Q* matrix). Then, the *Q* matrices of the plies are integrated by the three coupling matrices (known as *A*, *B*, *D* matrices). In order to calculate the stiffness of a composite laminate with a given layup of plies, CLT requires knowledge of the following four stiffness parameters of the uniaxial ply: longitudinal stiffness (*E*_*c*1_), transverse stiffness (*E*_c2_), shear modulus (G_*c12*_) and Poisson’s ratio (*ν*_*c*12_).

In the present study, *E*_*c*1_ was determined from the measured stiffness of the uniaxial laminate. The parameter *E*_*c2*_ was calculated by the inverse rule of mixtures model (Eq 6).

$$E_{c2}=\frac{1}{\frac{V_{f}}{E_{f2}}+\frac{V_{m}}{E_{m}}}$$ (6)

where *E*_*f2*_ is the transverse stiffness of the fibres, In a previous study of jute/epoxy composites by Cichocki and Thomason [8] values for *E*_*f1*_ (the longitudinal stiffness of fibres) and *E*_*f2*_ were found to be 39.4 and 5.5 GPa, respectively. A similar ratio between these two stiffness parameters is assumed in the present study. i.e. *E*_*f2*_ was estimated as equal to 5.5/39.4 × *E*_*f1*_. This ratio of 1/7 is supported by an almost identical ratio found by Baley et al. [14]. The parameter *G*_*c12*_ was calculated by the Halpin-Tsai equation [15] (Eq 7).

$$G_{c12}=\frac{G_{m}\left(1+\xi \eta V_{f}\right)}{\left(1-\eta V_{f}\right)}where\;\eta \;=\frac{\frac{G_{f}}{G_{m}}-1}{\frac{G_{f}}{G_{m}}+\xi }$$ (7)

where *ξ* is a fibre shape factor which was set to be 2 with the assumption that fibres have circular cross sections. The parameters *G*_*f*_ and *G*_*m*_ are the shear modulus of the fibres and the matrix, respectively. The matrix is assumed to be isotropic, and *G*_*m*_ can therefore be estimated as equal to *E*_m_/{2(1+*ν*_m_)}, where *ν*_*m*_ is the Poisson’s ratio of the matrix. In the above-mentioned study of jute/epoxy composites [8], the value for *G*_*f*_ was found to be 3.5 GPa. A similar ratio between the two stiffness parameters *G*_*f*_ and *E*_*f*_ was assumed in the present study, and *G*_*f*_ was accordingly estimated as equal to 3.5/39.4 × *E*_*f1*_.

Finally, the parameter *ν*_*c*12_ was estimated based on the findings in Cichocki and Thomason [8] where *ν*_*c*12_ was determined to be about 0.05 to 0.10 below *ν*_*m*_. Therefore, since *ν*_*m*_ in the present study was measured to be 0.35 for the epoxy resin, *ν*_*c*12_ for the composites was estimated to be 0.30.

In some cases, CLT calculations can be simplified under the assumptions of symmetry and balance of the plies in the composite. In the present study, however, such simplifications could not be done. The actual yarn orientation in the multiaxial composites, as measured by the FFO method, was used. The yarns in the composites were interpreted as being the “fibres” in the above presented description of the CLT calculations.

## Results and discussion

### Yarn orientation in fabrics

**Fig 7:**
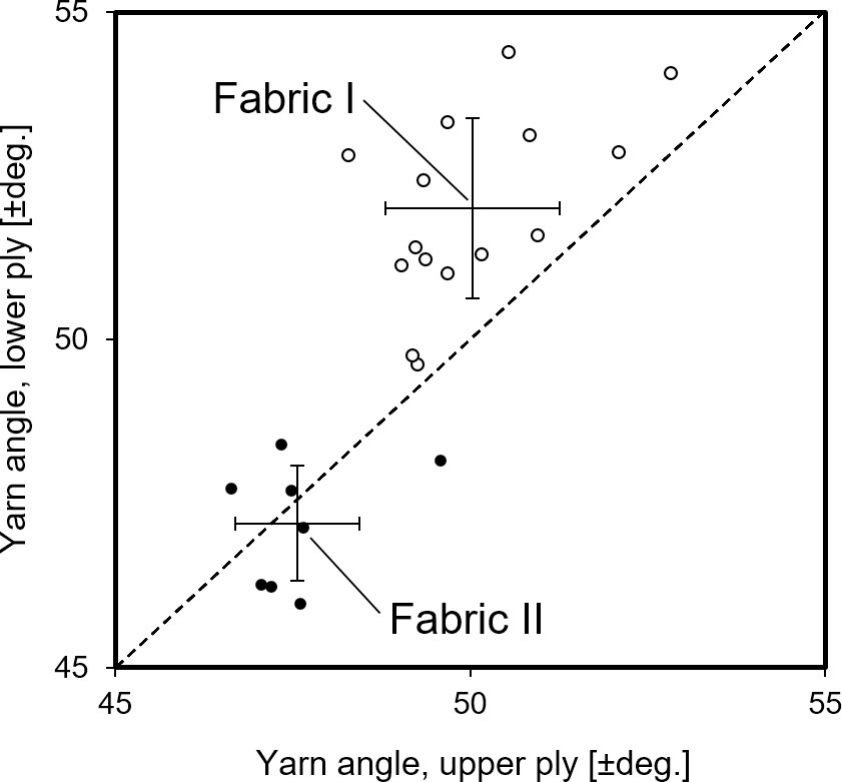
Relationship between measured yarn orientation angles in the lower and upper ply of the two types of biaxial flax fabrics. Crosses indicate means and standard deviations.

Fig 7 shows the results of the measured yarn orientations in the two types of biaxial fabrics. The diagram shows the relationship between the angles in the upper and lower ply. Each data point represents measurements by the FFO method of yarn angles in one fabric sheet. The dotted line in the diagram indicates the ideal *balanced* configuration of a fabric where the yarn orientation in the two plies are having equal + and–angles. It can be seen that for fabric I, all data points are above the line. The yarn angles for fabric I in the upper and lower ply are +50.0° ± 1.2° and -52.0° ± 1.4° (mean ± stdv.), respectively. Thus, fabric I is deviating from being balanced, and the yarn orientation can be given as +50°/-52°. For fabric II, the data points are distributed around the line. The yarn angles for fabric II in the upper and lower ply are +47.6° ± 0.9° and -47.2° ± 0.9° (mean ± stdv.), respectively. Thus, fabric II is almost balanced, and the yarn orientation can be given as +48°/-47°. The yarn angles in both fabrics, however, are larger than the nominal ones of +45° and -45°. Fig 4 shows an illustration of the resulting yarn directions of a multiaxial composite made with a balanced biaxial fabric where the + and–yarn angles are above 45°. Finally, in Fig 7, it can be observed that the scatter of yarn angles is lower for fabric II than for fabric I. Altogether, it is demonstrated that compared to fabric I, the yarn orientation in fabric II is (i) more balanced, (ii) closer to the nominal ± 45°, and (iii) more uniform. This is likely to be due to the presence of a stabilizing yarn in fabric II, leading to an improved quality of the fabric.

### Volumetric composition in composites

Table 2 shows the determined volumetric composition in the fabricated composite laminates. As can be observed, there is practically no difference in the volumetric composition of the multiaxial composites with the two types of fabrics (I and II) and with the two different layups (A and B). This demonstrates that the fabrics are of a constant quality, and that the applied fabrication technique is well controlled and repeatable. In the further analyses of composite properties, the overall average values of *V*_*f*_ = 31% and *V*_*m*_ = 69% will be used. Table 2 shows also the volumetric composition of the uniaxial composite laminate. Here *V*_*f*_ = 41% is larger than for the multiaxial laminates. This difference in fibre volume content can be explained by a difference in fibre packing ability of the two types of yarn assemblies [16, 17]. It is expected that for the same compaction pressure (in this case equal to the vacuum pressure of about 1 bar), the fibre packing ability of a filament wound uniaxial yarn assembly is larger than for a layup of multiaxial fabrics, and this leads to a higher fibre content in the former one. Thus, it is not readily possible to obtain the same fibre content in composite with the two types of yarn assemblies when the vacuum infusion technique is used. Finally, in Table 2, it can be observed that for all the fabricated composite laminates, the porosity content is below 1%, which means that the porosity content can be neglected in the further analyses [9].

**Table 2:** Volumetric composition of the fabricated multiaxial and uniaxial composite laminates.

Composite type	Layup	Weight fraction [%] (Mean ± stdv.)	Density [g/cm^3^] (Mean ± stdv.)	Volume fractions [%] (Mean ± stdv.)		
		*W*_*f*_	*ρ*_*c*_	*V*_*f*_	*V*_*m*_	*V*_*p*_
Multiaxial, fabric I	A	38.5 ± 0.4	1.273 ± 0.001	31.4 ± 0.3	68.6 ± 0.4	0.1 ± 0.2
	B	38.3 ± 0.9	1.272 ± 0.002	31.2 ± 0.7	68.8 ± 1.0	0.1 ± 0.3
Multiaxial, fabric II	A	38.4 ± 0.5	1.269 ± 0.005	31.6 ± 0.4	67.9 ± 0.7	0.0 ± 0.2
	B	37.5 ± 0.3	1.271 ± 0.002	30.9 ± 0.3	69.0 ± 0.2	0.1 ± 0.2
Uniaxial		48.4 ± 0.4	1.301 ± 0.002	40.9 ± 0.2	58.3 ± 0.3	0.8 ± 0.2

### Yarn orientation in composites

The measured yarn orientation in the tensile specimens of the multiaxial composites is presented in Table 3. The testing direction of the specimens (0, 45 or 90°) is used as the reference direction for the measured yarn angles. The schematic illustration in Fig 4 is a help in understanding the measured yarn angles with respect to the testing directions. Table 3 shows also the yarn orientation in the specimens calculated from the fabric, i.e. calculated from the measured yarn angles in the fabric (+50°/-52° and +48°/-47° for fabric I and II, respectively), taking into account the layup and the testing direction. As can be observed, the *measured* yarn angles in the tensile specimens compare well with the angles *calculated* from the fabric. The difference between the mean values is in the range 0–3 degrees, and in most cases the measured yarn angles in tensile specimens and the ones calculated from the fabric overlap each other within the range of standard deviations. Thus, altogether, it is demonstrated that the FFO method is a robust method, giving repeatable results, and it can be used both on fabrics and on composites.

**Table 3:** Measured yarn orientation in tensile specimens of the multiaxial composite laminates with fabric I and II. The testing direction of the specimens is used as the reference direction for the measured yarn angles (see Fig 4). Presented is also fabric based yarn orientation in the specimens calculated from the measured yarn orientation in the fabrics.

Fabric type	Layup type	Testing direction	Yarn orientation [deg.]				
				1^st^, 3^rd^ ply	2^nd^, 4^th^ ply	5^th^, 7^th^ ply	6^th^, 8^th^ ply
Fabric I	Layup A	0°	Specimens	52.8 ± 0.8	-49.5 ± 0.2	-38.4 ± 0.5	39.2 ± 0.6
			Fabric	52.0 ± 1.4	-50.0 ± 1.2	-38.0 ± 1.4	40.0 ± 1.2
		45°	Specimens	-83.3 ± 0.6	-6.3 ± 0.8	6.9 ± 1.0	84.6 ± 1.7
			Fabric	-83.0 ± 1.4	-5.0 ± 1.2	7.0 ± 1.4	85.0 ± 1.2
		90°	Specimens	-36.6 ± 0.9	36.9 ± 0.2	50.2 ± 0.6	-48 ± 0.2
			Fabric	-38.0 ± 1.4	40.0 ± 1.2	52.0 ± 1.4	-50.0 ± 1.2
	Layup B	0°	Specimens	52.8 ± 0.1	-49.3 ± 0.6	53.0 ± 0.6	-49.5 ± 0.3
			Fabric	52.0 ± 1.4	-50.0 ± 1.2	52.0 ± 1.4	-50.0 ± 1.2
		45°	Specimens	-80.4 ± 1.3	-6.0 ± 0.4	-82.4 ± 1.4	-6.2 ± 0.2
			Fabric	-83.0 ± 1.4	-5.0 ± 1.2	-83.0 ± 1.4	-5.0 ± 1.2
		90°	Specimens	-37.3 ± 0.8	38.7 ± 0.7	-38.4 ± 0.4	37.6 ± 0.4
			Fabric	-38.0 ± 1.4	40.0 ± 1.2	-38.0 ± 1.4	40.0 ± 1.2
Fabric II	Layup A	0°	Specimens	47.0 ± 1.1	-44.8 ± 1.0	-43.1 ± 0.6	40.7 ± 0.9
			Fabric	47.6 ± 0.9	-47.2 ± 0.9	-42.4 ± 0.9	42.8 ± 0.9
		45°	Specimens	-86.3 ± 1.2	-0.6 ± 0.3	1.4 ± 0.8	84.6 ± 1.5
			Fabric	-87.4 ± 0.9	-2.2 ± 0.9	2.6 ± 0.9	87.8 ± 0.9
		90°	Specimens	-42.1 ± 1.1	43.7 ± 1.0	46.4 ± 0.7	-48.7 ± 1.0
			Fabric	-42.4 ± 0.9	42.8 ± 0.9	47.6 ± 0.9	-47.2 ± 0.9
	Layup B	0°	Specimens	46.1 ± 2.2	-45.6 ± 0.2	45.6 ± 1.7	-46.7 ± 1.2
			Fabric	47.6 ± 0.9	-47.2 ± 0.9	47.6 ± 0.9	-47.2 ± 0.9
		45°	Specimens	-88.0 ± 1.2	-2.3 ± 1.2	-86.2 ± 0.7	-3.8 ± 1.0
			Fabric	-87.4 ± 0.9	-2.2 ± 0.9	-87.4 ± 0.9	-2.2 ± 0.9
		90°	Specimens	-43.7 ± 0.7	40.5 ± 0.6	-39.9 ± 0.7	40.3 ± 0.7
			Fabric	-42.4 ± 0.9	42.8 ± 0.9	-42.4 ± 0.9	42.8 ± 0.9

### Tensile properties of composites

**Fig 8:**
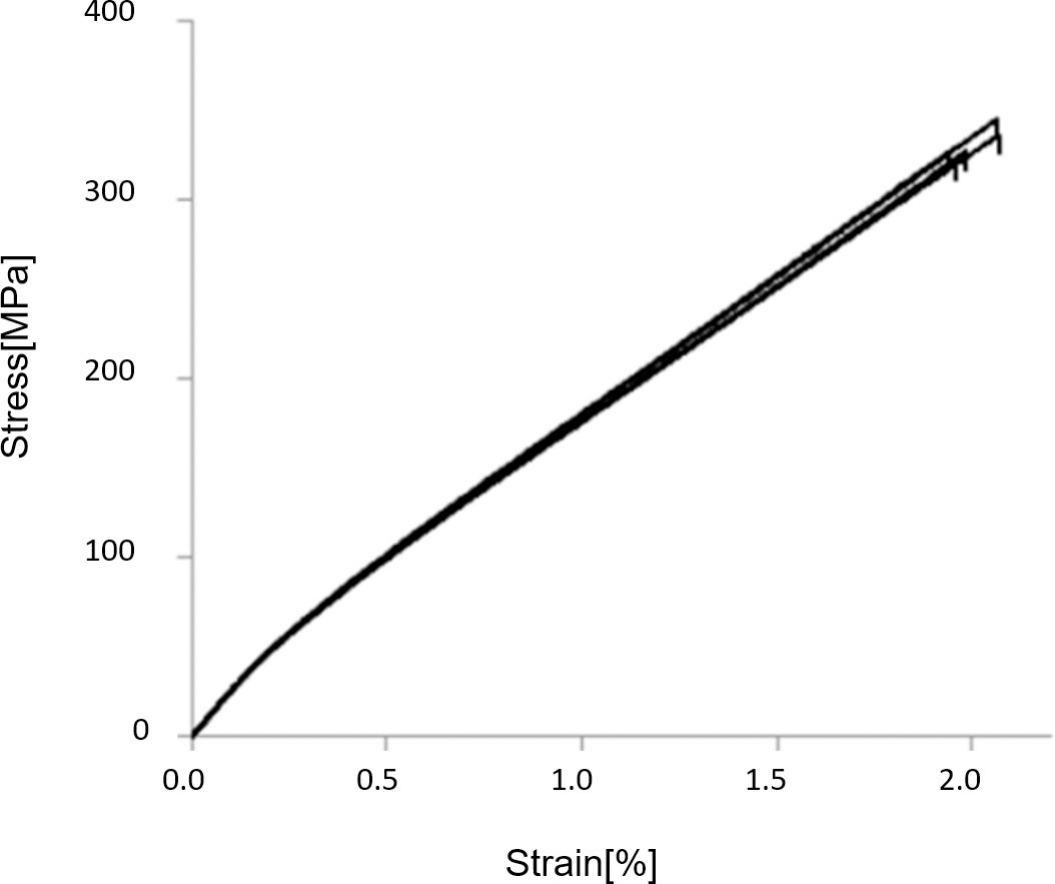
Measured stress-strain curves of the uniaxial flax fibre composites.

The measured stress-strain curves of the uniaxial composites are shown in Fig 8. The curves can be seen to have a bi-linear shape which is typical for uniaxial natural fibre composites [18]. The transition between the two linear parts is taken place at a strain value of about 0.20%. In the present study, stiffness is determined in the first linear part, in the strain interval 0.01–0.15%. Stiffness, strength, and failure strain of the uniaxial composites is determined to be 25.0 ± 0.3 GPa, 331 ± 9 MPa, and 2.0 ± 0.1%, respectively. By using the rule of mixtures model, the stiffness of the flax yarn can be back-calculated to be 57 GPa, which is comparable to a back-calculated value of 66 GPa found in a previous study of similar flax yarn composites [19]. In these calculations, the off-axis twisting angle of the fibres in the yarn (on about 15°) is not taking into account, and the back-calculated value represents therefore the effective stiffness of the flax yarn in the composites. Thus, the stiffness of the flax fibres themselves is expectedly larger than the back-calculated value.

In the CLT model predictions, the stiffness of the uniaxial composites is used as input data to predict the stiffness of the multiaxial composites. However, since *V*_*f*_ is not equal in the uniaxial and the multiaxial composites (with values of 41 and 31%, respectively), the stiffness of the uniaxial composites needs to be corrected to account for this. By using the rule of mixtures model, a stiffness on 19.8 GPa is calculated for the uniaxial composites with *V*_*f*_ = 31%.

**Fig 9:**
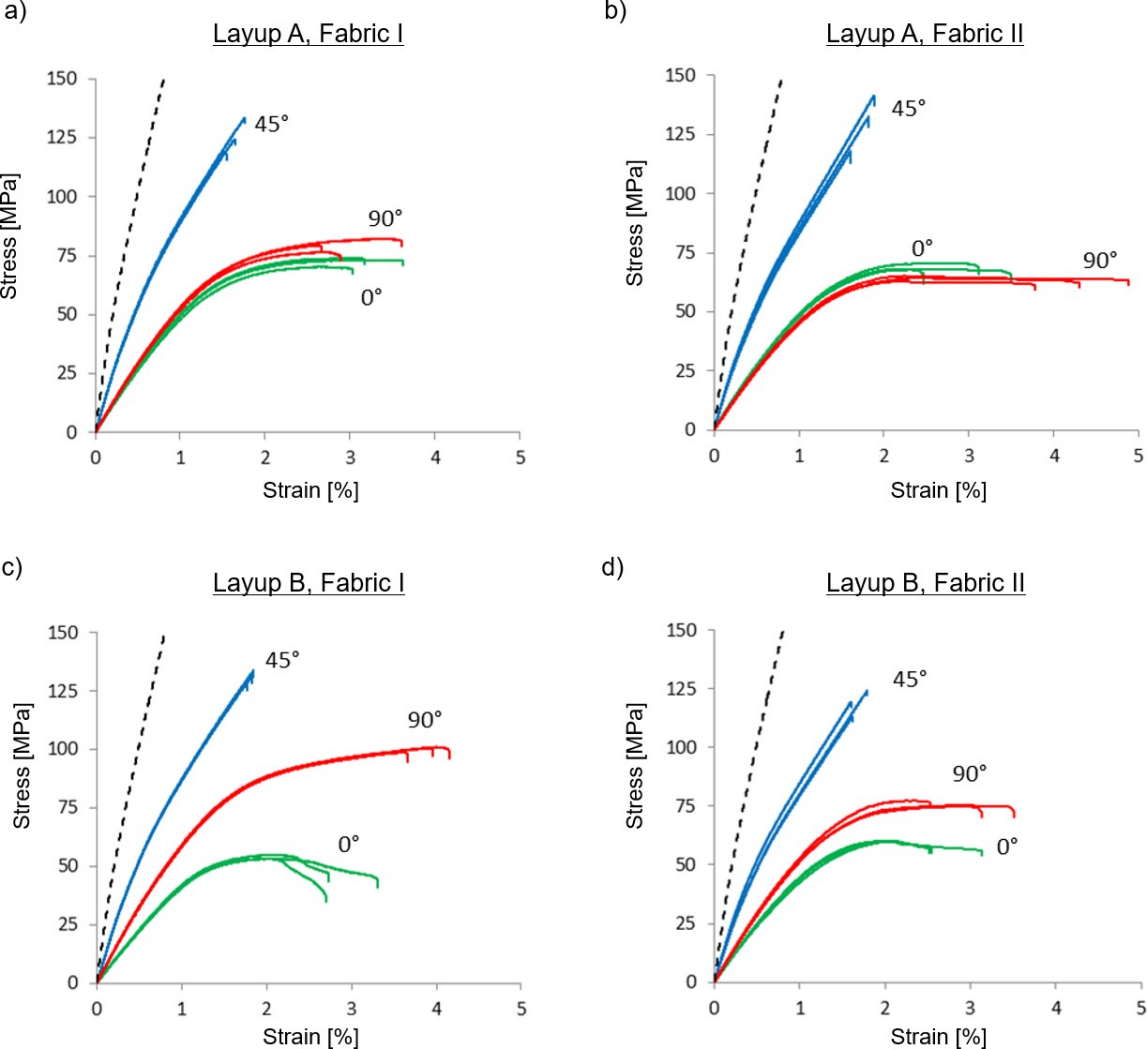
Measured stress-strain curves of the multiaxial flax fibre composite laminates with layup A: (a) fabric I and (b) fabric II, and with layup B: (c) fabric I and (d) fabric II. Shown are stress-strain curves for the three testing directions of 0°, 45° and 90°. For comparison, the initial part of the stress-strain curve of the uniaxial composite laminate is shown by a dotted line.

The measured stress-strain curves of the multiaxial composites are shown in Fig 9. For layup A, the stress-strain curves in the 45° testing direction are much above the curves in the 0° and 90° directions, and the curves in the latter two directions are overlying each other. This is expected from the orientation of the yarns in the multiaxial composites with layup A, as illustrated in Fig 4. In the 45° direction, the yarns in two out of the four groups of plies are placed with low off-axis angles on both side of the testing direction. In the 0° and 90° directions, the yarns in all four groups of plies are placed with large off-axis angles, and they are forming equal off-axis angles in the two testing directions. For layup B, the stress-strain curves in the 45° direction are again, as expected, much above the curves in the 0° and 90° directions. However, now the curves in the 0° and 90° directions are clearly separated; the curves in the 90° direction are above the curves in the 0° direction. Again, this is to be expected from the orientation of the yarns in the multiaxial composites with layup B, as illustrated in Fig 4. In the 90° testing direction, the yarns in the two groups of plies are placed with off-axis angles below 45°, whereas in the 0° testing direction, the yarns in the two groups of plies are placed with off-axis angles above 45°. Moreover, for layup B, the separation of stress-strain curves in the 0° and 90° directions is larger for composites with fabric I than for composites with fabric II. This is due to the fact that the yarn orientation in fabric I, as compared to fabric II, is less balanced and further away from the nominal ± 45° (see Fig 7). The effect of this on the separation of stress-strain curves in the 0° and 90° directions can also be understood from Fig 4.

With respect to the shape of the stress-strain curves for the multiaxial composites shown in Fig 9, it can be observed that the transition point between the linear parts of the curves is different for the different testing directions, and it also differs from the one found for the uniaxial composites. The strain values of the transition point are about 0.50 and 1.50% for the 45° and 0°/90° directions, respectively, compared to the value of 0.20% for the uniaxial composites.

The present study will focus on the stiffness of the multiaxial composites, i.e. the slope of the first linear part of the stress-strain curves. Other characteristics of the stress-strain curves, e.g. the transition point, the strength, and the failure strain will not be analysed further. In a recent study by Koh and Madsen [20], the strength of multiaxial flax fibre composites, manufactured from the same flax fabric (type I) as used in the present study, is predicted by applying failure criteria theories of composites.

Table 4 shows the measured stiffness of the multiaxial composites. The values follow the trends presented above for the stress-strain curves. Testing in the 45° direction gives the largest stiffness of the composites, with values in the range 11.6–12.5 GPa. In the 0° and 90° directions, stiffness is almost halved with values in the range 4.6–6.9 GPa. For layup A, there is no large difference in stiffness between the 0° and 90° directions. On the other hand, for layup B, there is a clear difference in stiffness between the 0° and 90° directions, with values of 4.6 and 6.9 GPa for fabric I, and 5.1 and 6.1 GPa for fabric II, respectively. Qualitatively speaking, these differences in stiffness are well expected from the differences in off-axis angles between the testing directions, as already explained above. Next, a quantitative correlation will be established for stiffness of the multiaxial composites by using CLT predictions.

**Table 4:** Measured stiffness of the multiaxial flax fibre composite laminates.

Layup type	Testing direction	Stiffness [GPa]	
		Fabric I	Fabric II
Layup A	0°	5.7 ± 0.1	5.7 ± 0.1
	45°	12.5 ± 0.1	12.2 ± 0.4
	90°	6.1 ± 0.1	5.3 ± 0.1
Layup B	0°	4.6 ± 0.1	5.1 ± 0.1
	45°	12.2 ± 0.1	11.6 ± 0.5
	90°	6.9 ± 0.1	6.1 ± 0.1

### Prediction of composite stiffness

In order to predict the stiffness of a multiaxial composite with a given layup of plies, CLT requires knowledge of four stiffness parameters of the uniaxial ply: longitudinal stiffness (*E*_*c*1_), transverse stiffness (*E*_c2_), shear modulus (G_*c12*_) and Poisson’s ratio (*ν*_*c*12_). In the present case of the uniaxial flax fibre composite, the longitudinal stiffness is calculated to be 19.8 GPa, by correcting the measured stiffness on 25.0 GPa to a V_f_ on 31%. The transverse stiffness is calculated to be 3.7 GPa, by using Eq 6 with a transverse fibre stiffness on 7.9 GPa. The shear modulus is calculated to be 1.7 GPa, by using Eq 7 with a shear fibre stiffness on 5.0 GPa and shear matrix stiffness on 1.1 GPa. Finally, the Poisson’s ratio is estimated to be 0.30. Further applied input to the CLT predictions is the yarn orientation in the biaxial flax fibre fabric, which was measured by the FFO method.

**Fig 10:**
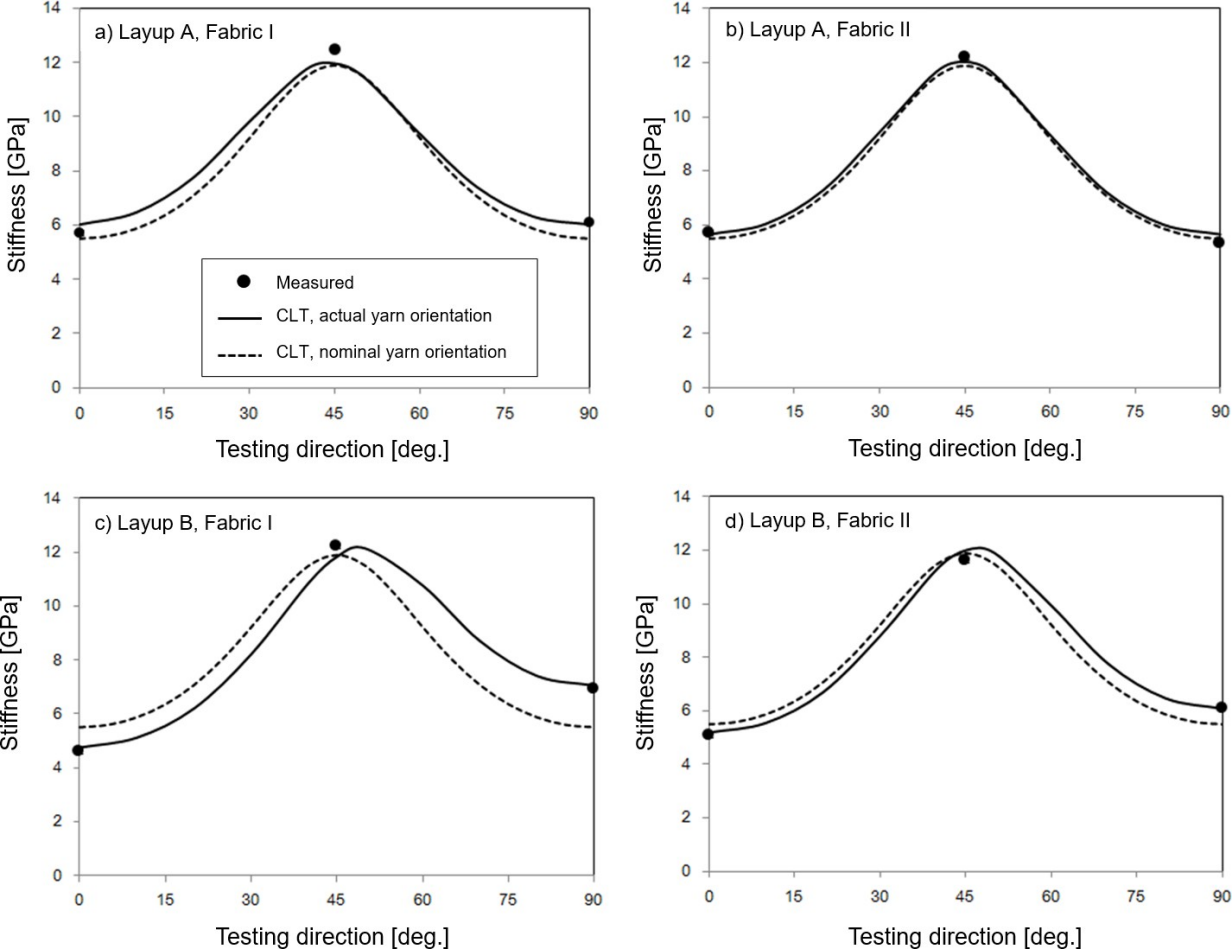
Relationship between stiffness and testing direction of the multiaxial flax fibre composite laminates with layup A: (a) fabric I and (b) fabric II, and with layup B: (c) fabric I and (d) fabric II. Data points are measured stiffness. Full lines are CLT predictions using the actual yarn orientation in the composites, as measured by the FFO method. Dotted lines are CLT predictions using a nominal yarn orientation on ±45° in the composites.

Fig 10 shows the relationship between the CLT predictions and the testing direction of the multiaxial composite laminates, together with the measured stiffness. Overall, the CLT predictions are in good agreement with the measured stiffness at the three testing directions (0°, 45° and 90°). The deviations between predictions and measurements are in the range 0–0.7 GPa. Thus, it is demonstrated that stiffness of multiaxial flax fibre composites can be accurately predicted by CLT, without any fitting constants, but based on independently determined stiffness parameters of the related uniaxial flax fibre composite using the presented methodology of micromechanical models. In addition, CLT requires information about the yarn orientation in the multiaxial composites.

Fig 10 shows two curves for the CLT predictions; one curve is using the *actual yarn orientation* (based on fabric measurements using the FFO method), and the other curve is using the *nominal yarn orientation* of ± 45°. The nominal curve is the same in the four diagrams. Although the actual and nominal curves are close to each other, it can be observed that in general, the actual curves are in better agreement with the measured stiffness of the composites than the nominal curves. The mean deviation between the predicted and measured stiffness is 0.2 GPa for the actual curves (corresponding to 3% relative deviation), and 0.5 GPa for the nominal curves (corresponding to 8% relative deviation). The deviations show also some larger extremes for the nominal curves than for the actual curves, e.g. for layup B and fabric I, the deviations for the nominal curves are 0.9 and 1.4 GPa for the testing directions 0° and 90°, respectively (corresponding to about 20% relative deviation). In general, it can be seen that the nominal curves deviate more from the actual curves for fabric I than for fabric II, and for layup B than for layup A.

Altogether, it is demonstrated that measurements of actual yarn orientations lead to better predictions of stiffness of multiaxial composites, and the significance is larger for fabrics which deviate more from the nominal yarn orientations (fabric I), and for layups which potentially are leading to large deviations in yarn orientations (layup B). In a practical context, the benefit of the Fast Fibre Orientation method must be considered with respect to the involved effort for performing the measurements, and the assurance for getting accurate predictions for all fabrics and layups.

## Conclusions

The present study is addressing prediction of stiffness of multiaxial flax fibre composite laminates by classical laminate theory (CLT). Multiaxial flax fibre composites are fabricated with two types of biaxial non-crimp fabrics, having a nominal yarn orientation of ±45°. Uniaxial flax fibre composites are fabricated to provide input parameters for the CLT predictions of stiffness of the multiaxial composites. The fabricated composites are characterised by volumetric composition, yarn orientation and tensile properties.

A fast and easy operational Fast Fibre Orientation (FFO) method is developed to determine the actual yarn orientation in fabrics and composites. The method is based on a standard flat head scanner and utilises 2D Fast Fourier Transformation theory. The yarn orientation in the two biaxial fabrics is measured to be +50/-52° and +48/-47°. The yarn orientation in the later fabric is closer to the nominal ± 45°, more balanced, and more uniform. The measured yarn angles in the composites are found to compare well with the angles determined from the fabrics, with a difference in the range 0–3 degrees. Altogether, it is demonstrated that the FFO method is a robust method, giving repeatable results, and it can be used both on fabrics and composites.

Stiffness of the uniaxial flax fibre composites with a fibre content of 41% is measured to be 25.0 GPa. The effective stiffness of the flax yarn is back-calculated to be 57 GPa. The determined values of the required four input parameters for the CLT predictions are 19.8 GPa for longitudinal stiffness, by correcting the measured stiffness on 25.0 GPa to a fibre content on 31%, 3.7 GPa for transverse stiffness, 5.0 GPa for shear stiffness, and 0.30 for Poisson’s ratio.

Stiffness of the multiaxial flax fibre composites is measured in the three testing directions of 0°, 45° and 90°. As expected from the biaxial yarn orientation in the composites, testing in the 45° direction gives the largest stiffness of the composites, with values in the range 11.6–12.5 GPa. In the 0° and 90° directions, stiffness is almost halved with values in the range 4.6–6.9 GPa.

CLT predictions are shown to be in good agreement with the measured stiffness of the multiaxial flax fibre composites at the three testing directions. The use of the actual yarn orientations measured by the FFO method, instead of the nominal yarn orientations of ±45°, is shown to result in improved CLT predictions of stiffness with a mean deviation between predictions and measurements on 0.2 GPa. Altogether, it is demonstrated that stiffness of multiaxial flax fibre composites can be accurately predicted by CLT, without any fitting constants, based on independently determined stiffness parameters of the related uniaxial flax fibre composite, and based on measured yarn orientations in the flax fabric.

## Supporting information

S1 Table Raw test data for Fig 8. (XLSX)

S2 Table Raw test data for Fig 9a) and 9b). (XLSX)

S3 Table Raw test data for Fig 9c) and 9d). (XLSX)

S4 Table Raw measurement data for Fig 7. (XLSX)

S5 Table Raw measurement data for Table 3. (XLSX)
